# Aberrant ALPP Expression Serves as a Prognostic Biomarker and Facilitates Cholangiocarcinoma Progression through Immune Evasion and PI3K-Akt Signaling Activation

**DOI:** 10.7150/ijms.116260

**Published:** 2025-10-01

**Authors:** Guo-Wei Wu, Yi-Chung Chien, Li-Yuan Bai, Yung-Luen Yu

**Affiliations:** 1 Graduate Institute of Biomedical Sciences, China Medical University, Taichung 406040, Taiwan.; 2 Institute of Translational Medicine and New Drug Development, China Medical University, Taichung 406040, Taiwan.; 3 Center for Molecular Medicine, China Medical University Hospital, Taichung 404327, Taiwan.; 4 Department of Hematology and Oncology, China Medical University Hospital, Taichung 404327, Taiwan.; 5 Cancer Biology and Precision Therapeutics Center, China Medical University, Taichung 406040, Taiwan.; 6 Office of Research and Development, Asia University, Taichung 41354, Taiwan.

**Keywords:** cholangiocarcinoma, ALPP, CA19-9, tumor-infiltrating immune cells, PI3K-Akt pathway

## Abstract

Cholangiocarcinoma (CCA) is a highly aggressive malignancy and represents the most common form of adenocarcinoma in the hepatobiliary system. Placental alkaline phosphatase (ALPP), a member of the alkaline phosphatase (ALP) isoenzyme family, catalyzes phosphate ester hydrolysis under alkaline conditions. While ALPP overexpression has been observed in various germ cell tumors and specific cancers, its functional relevance and regulatory mechanisms in CCA remain poorly understood. In this study, we evaluated *ALPP* expression in CCA patient cohorts and explored its correlation with clinicopathological features and patient prognosis. We further assessed the relationship between *ALPP* expression and tumor-infiltrating immune cells, focusing on B cells and dendritic cells (DCs). To elucidate *ALPP*-associated molecular networks, weighted gene co-expression network analysis (WGCNA) was performed, followed by functional enrichment analyses using Gene Ontology (GO) and the Kyoto Encyclopedia of Genes and Genomes (KEGG) pathways. The methylation landscape of the ALPP gene was also examined. Our findings demonstrated that elevated *ALPP* expression is significantly associated with increased serum CA19-9 levels and reduced overall survival in CCA patients. Immune infiltration analyses revealed a positive correlation between ALPP expression and the abundance of infiltrating B cells and DCs. WGCNA identified a gene module associated with *ALPP* that was highly enriched in the PI3K-Akt signaling pathway. Additionally, hypomethylation of a specific CpG site (cg19654061) within the ALPP gene was significantly associated with its upregulation. Collectively, these results suggest that ALPP functions as a potential prognostic biomarker in CCA and may contribute to disease progression through modulation of the immune microenvironment and activation of oncogenic signaling pathways.

## 1. Introduction

Cholangiocarcinoma (CCA) is a highly lethal and heterogeneous malignancy that arises from the epithelial cells of the biliary tree and belongs to the group of adenocarcinomas. Based on the primary anatomical site of origin, CCA is classified into three subtypes: intrahepatic cholangiocarcinoma (iCCA), which arises within the liver parenchyma above the second-order bile ducts; perihilar cholangiocarcinoma (pCCA), which occurs at the hepatic hilum; and distal cholangiocarcinoma (dCCA), which originates in the common bile duct below the insertion of the cystic duct. Notably, many databases categorize both pCCA and dCCA as extrahepatic CCA 1-3.

In addition, each subtype of cholangiocarcinoma exhibits distinct genetic alterations, clinical features, and therapeutic approaches. Treatment options for CCA include surgical resection, chemotherapy, radiotherapy, targeted therapy, immunotherapy, and transarterial chemoembolization (TACE), among others. Currently, early surgical resection or TACE is considered the primary first-line treatment strategy. However, most patients are diagnosed at an advanced stage, rendering them ineligible for curative interventions 4, 5.

For advanced-stage CCA, treatment typically involves second-line chemotherapy regimens, such as the combination of gemcitabine and cisplatin. In the third line setting, targeted therapies like Pemigatinib, an FGFR2 inhibitor, and Bevacizumab (Avastin), a vascular endothelial growth factor (VEGF) inhibitor, are utilized, alongside immunotherapies such as PD-1 inhibitors, which act as immune checkpoint inhibitors. Despite significant progress in molecular medicine, pathological diagnosis, and treatment strategies for CCA, these advancements have not kept pace with the increasing incidence and mortality rates of the disease. The heterogeneity of the tumor and its often-subtle early symptoms contribute to a diagnosis frequently made at an advanced stage. Consequently, the efficacy of current treatment modalities remains limited, leading to a poor prognosis for patients with CCA 4, 6.

The 5-year survival rate for patients with CCA ranges from 7% to 20%, and the rate of tumor recurrence following surgical resection remains notably high, which is a significant challenge in the management of this disease 7. Epidemiological studies investigating the etiology of CCA commonly classify the disease into two categories: fluke-related and non-fluke-related CCA. In Southeast Asia, the majority of CCA cases are associated with liver fluke infections. In contrast, in other regions, research has highlighted stronger associations between intrahepatic cholangiocarcinoma (iCCA) and conditions such as primary sclerosing cholangitis, cirrhosis, non-alcoholic fatty liver disease (NAFLD), and hepatitis B 8. Additionally, choledocholithiasis (bile duct stones) has been more strongly linked to perihilar (pCCA) and/or distal (dCCA) cholangiocarcinoma 9, 10.

While multiple risk factors contribute to the development of CCA, no single, definitive risk factor has been identified. Pathological and molecular studies have revealed considerable variations in the etiology of CCA, as demonstrated by comprehensive genomic and epigenomic profiling. These studies suggest that different subtypes of cancer within the same organ can arise through distinct extrinsic and intrinsic carcinogenic mechanisms 11-13. Furthermore, CCA tumors are characterized by elevated levels of DNA methylation. Integrative analyses of somatic mutations and DNA methylation have indicated that tumor initiation involves not only genetic driver mutations but also concurrent epigenetic modifications 9, 12. From a metabolic perspective, CCA is associated with significant alterations, including an increase in glycolysis and changes in glycogen homeostasis, which support rapid tumor cell proliferation 14. Thus, investigating the complex interactions between genetic variations, metabolic shifts, and CCA pathogenesis is essential for advancing predictive models and therapeutic approaches.

Alkaline phosphatase (ALP) is a group of essential enzymes that catalyze the hydrolysis of phosphate esters under alkaline conditions, thereby releasing inorganic phosphate 15, 16. ALP is widely distributed across various human tissues, including the liver, bone, intestine, and placenta. It plays a critical role in bone development and mineralization during childhood and adolescence and participates in nutrient absorption and metabolism within the intestinal tract. Clinical studies have demonstrated that abnormal serum ALP levels are associated with a variety of pathological conditions 17, 18. Notably, elevated ALP concentrations are often observed in cases of cancer, liver injury, and bone metastasis, reflecting tissue damage or increased tumor cell activity. As a result, serum ALP has been widely adopted as a biomarker for clinical diagnosis and prognostic evaluation 19, 20.

ALP is composed of multiple isoenzymes, each exhibiting distinct tissue-specific expression. To date, four major ALP isoenzymes have been identified: intestinal (ALPI), placental (ALPP), germ cell (ALPG), and tissue-nonspecific (ALPL). Among these, ALPL is predominantly expressed in the liver, bone, and kidney, and represents the primary source of ALP in the serum 20. These isoenzymes differ in their enzymatic properties, gene regulation, and associations with various diseases. Clinically, analyzing the proportions and variations of different ALP isoenzymes can help elucidate the origin and potential causes of elevated ALP levels, providing valuable insights particularly in the differential diagnosis of hepatic versus skeletal metastases 21. Placental alkaline phosphatase (ALPP, also known as PLAP) has long been recognized as a tumor biomarker in germ cell tumors such as seminomas 21. Aberrant re-expression of ALPP has also been observed in somatic malignancies. Notably, Chen et al. reported that ALPP is upregulated in certain hepatocellular carcinomas with increased tumor cell motility, suggesting a pro-metastatic phenotype 21. Consistently, a recent pan-cancer analysis found >2-fold ALPP overexpression in multiple solid tumors (e.g., ovarian, endometrial carcinomas) relative to normal tissues, and linked high ALPP levels to aggressive disease courses in at least one cancer type (pancreatic adenocarcinoma) 22. These findings underscore the potential pro-tumorigenic functions of ALPP across cancers and support our investigation of its role in cholangiocarcinoma.

Notably, ALPP, in addition to its high expression in the placenta during pregnancy, has been found to be aberrantly upregulated in various germ cell tumors and specific types of cancer. Recent studies have shown that *ALPP* is overexpressed in hepatocellular carcinoma and is associated with enhanced cellular motility, suggesting a potential pro-tumorigenic role. Moreover, *ALPP* may also be involved in modulating both innate and adaptive immune responses in immune-related diseases. For instance, in animal models with *ALPP* overexpression, increased sensitivity to lipopolysaccharide (LPS)-induced septic shock and reduced immune rejection in allogeneic skin grafts have been observed, indicating the immunomodulatory potential of *ALPP*. These findings offer new insights into the role of ALP isoenzymes in tumor immunity, metastasis, and host response, further supporting their clinical relevance as potential diagnostic and therapeutic targets 21.

Although ALP has increasingly been recognized as a serum biomarker for cancer diagnosis in clinical practice, most studies have focused primarily on its application in blood-based detection. Investigations into its biological functions and underlying molecular mechanisms in cancer progression remain limited. There is a lack of comprehensive and systematic research on the role of *ALPP* in CCA. Existing research provides insufficient evidence regarding the involvement of *ALPP* in CCA tumor development, metastasis, and its interaction with the tumor immune microenvironment, highlighting a significant gap in current knowledge. Therefore, elucidating the oncogenic role of *ALPP* in CCA is of great scientific interest, as it may not only enhance our understanding of its functional contribution to tumor progression but also facilitate the identification of novel prognostic biomarkers and therapeutic targets, ultimately advancing the diagnosis and treatment strategies for cholangiocarcinoma.

In this study, we systematically analyzed the association between *ALPP* expression and various clinical characteristics, as well as survival outcomes, in patients with CCA, utilizing data from The Cancer Genome Atlas (TCGA) via the UCSC Xena platform. In addition, we examined the methylation status of the *ALPP* promoter across different tissue groups and expression levels. We further explored the relationship between *ALPP* expression and tumor-infiltrating immune cells within the CCA tumor microenvironment (TME. Gene Ontology (GO) and Kyoto Encyclopedia of Genes and Genomes (KEGG) enrichment analyses indicated significant activation of the PI3K-Akt signaling pathway. To the best of our knowledge, this study is the first to comprehensively characterize the clinical relevance, epigenetic regulation, and immunological implications of *ALPP* in cholangiocarcinoma, thereby providing novel insights into its potential role in CCA pathogenesis.

## 2. Materials and Methods

### 2.1 ALPP expression and overall survival analysis by UCSC Xena

The mRNA expression levels of *ALPP* were analyzed using the UCSC Xena browser (http://xena.ucsc.edu), an open-access web-based platform for exploring functional genomics data. Expression data were obtained from the TCGA-CHOL database, consisting of 45 samples in total, including solid tissue normal samples and CCA tumor samples. Gene expression values were presented as log₂(x+1)-transformed RSEM-normalized counts, representing gene-level transcription estimates 23-25. Based on the distribution of *ALPP* expression across all samples, CCA patients were stratified into two groups: a high-expression group (RSEM values above the upper quartile, n = 9) and a low-expression group (RSEM values below the upper quartile, n = 36). Patients were stratified into ALPP-high and ALPP-low groups based on the median ALPP mRNA expression level in the cohort. Kaplan-Meier survival analysis was subsequently performed to evaluate the association between *ALPP* expression and overall survival in CCA patients.

### 2.2. Clinic data analysis and DNA methylation of the ALPP gene

Using an open website, UALCAN (https://ualcan.path.uab.edu/index.html) 24, to extract the clinic and DNA methylation data of the TCGA-CHOL project. Correlations between patient prognosis and various clinicopathological parameters, including Age, Gender, *ALPP* expression, Tumor location, Serum CA19-9 levels, and Clinical stage, were systematically evaluated. DNA methylation data were obtained using the Illumina Infinium HumanMethylation450 platform. The methylation status of individual CpG sites within the *ALPP* gene was analyzed in relation to tissue type (normal vs. tumor) and *ALPP* expression levels. All analyses were performed using the RStudio.

### 2.3. TIMER database to estimate tumor-infiltrating immune cells

The TIMER database (https://cistrome.shinyapps.io/timer/), which comprises 10,897 samples across 32 cancer types from TCGA, serves as a comprehensive and widely utilized resource for estimating the abundance of tumor-infiltrating immune cells, including B cells, CD4⁺ T cells, CD8⁺ T cells, neutrophils, macrophages, and dendritic cells. 26 In this study, we utilized the Gene module within the TIMER platform to investigate the correlation between *ALPP* expression and the infiltration levels of various immune cell types.

### 2.4. Weighted gene co-expression network analysis (WGCNA)

We performed WGCNA to explore gene-gene interactions and their associations with clinical traits, particularly in the context of high versus low ALPP expression groups. RNA-seq data were analyzed following the methodology established by Langfelder et al., enabling the identification of ALPP-related gene modules and their potential functional relevance 27.

### 2.5. Gene Ontology (GO), Kyoto Encyclopedia of Genes and Genomes (KEGG)

Computational analyses were conducted using R Studio (version 4.4.1) to assess the statistical significance of biological signaling pathways. GO and KEGG enrichment analyses were performed for ALPP-related genes and key genes identified through WGCNA, utilizing the clusterProfiler package. Pathway enrichment analysis was conducted with GSEA to identify activation of oncogenic pathways such as PI3K-Akt 28.

### 2.6. Statistical analysis

Multivariate Cox proportional hazards regression analysis was performed to assess the prognostic impact of *ALPP* expression in conjunction with other clinicopathological variables. All statistical analyses were conducted using R software (version 4.4.1), with relevant packages including survminer, forestplot, pheatmap, ggplot2, clusterProfiler, enrichplot, WGCNA. A two-tailed p-value < 0.05 was considered statistically significant.

## 3. Results

### 3.1. High *ALPP* expression and poor prognosis in cholangiocarcinoma

**Figure 1:**
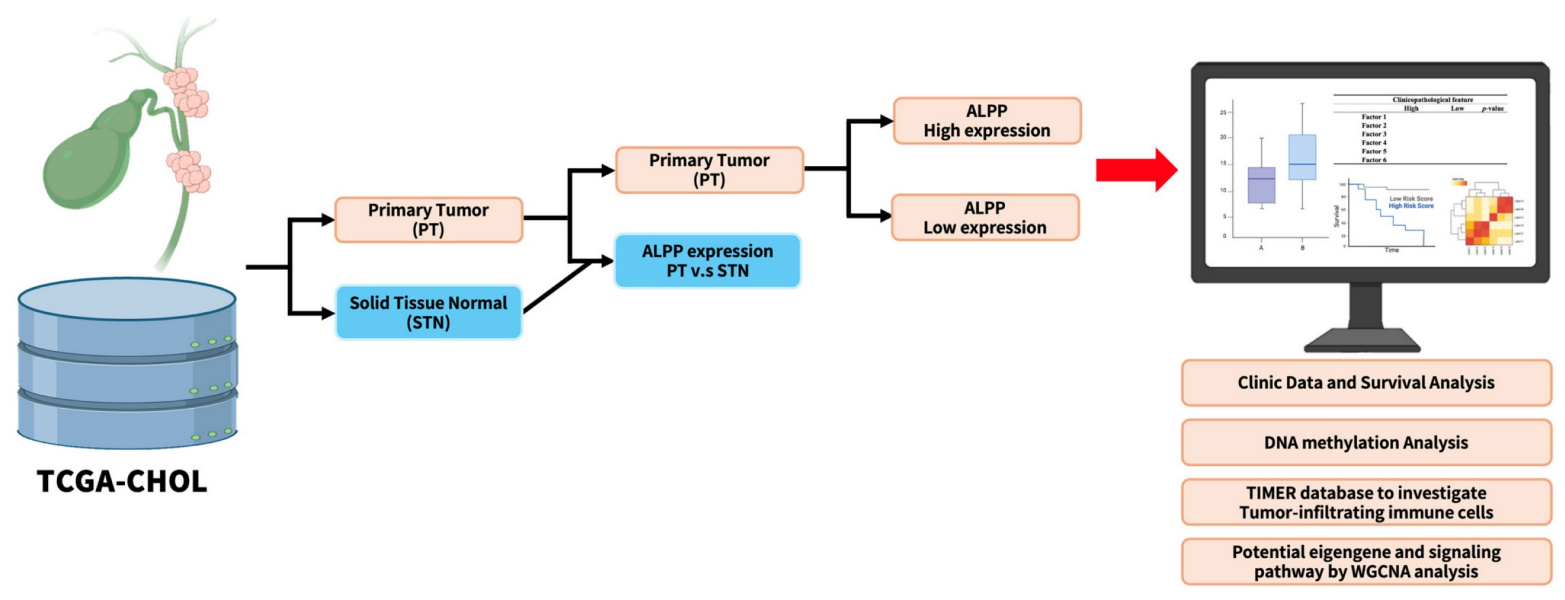
Workflow of the study analysis process. Workflow creation was performed using Biorender (https://app.biorender.com).

**Figure 2:**
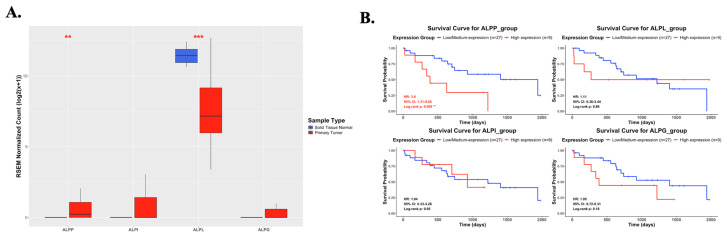
Differential mRNA expression of *ALPP* and other *ALP* isoenzymes between normal and CCA samples and their association with overall survival in CCA. (A) mRNA expression levels of *ALPP* and other *ALP* isoenzymes in normal (n = 9) and CCA tissues (n = 36) from the UCSC Xena database (https://xenabrowser.net/datapages/). (B) Kaplan-Meier survival curves illustrating overall survival in CCA patients with high (red) and low (blue) expression for *ALPP* and other ALP isoenzymes. Mean ± SD, ** < 0.01, *** <0.001.

As showed in Figure 1, this study was conducted to investigate the clinical relevance of *ALPP* in CCA. Analysis of the TCGA-CHOL dataset, accessed through the UCSC Xena platform, demonstrated that CCA patients had significantly higher mRNA expression of *ALPP* and lower expression of *ALPL*, when compared to normal controls, among various alkaline phosphatase (*ALP*) isoenzymes (Figure 2A). Notably, Kaplan-Meier survival analysis revealed that elevated *ALPP* expression was the only *ALP* isoenzyme significantly associated with poorer prognosis in CCA patients (Figure 2B). Clinical risk factor analysis identified CA19-9, a critical tumor marker for digestive cancers, as significantly elevated in patients with high *ALPP* expression (Table 1). Additionally, multivariate Cox proportional hazards regression analysis confirmed the significant association between *ALPP* expression and patient outcomes (Table 2). These results suggest that *ALPP* might play a pivotal role in the pathogenesis of CCA through potential molecular interactions.

### 3.2. Correlation of *ALPP* and immune cell infiltration

**Figure 3:**
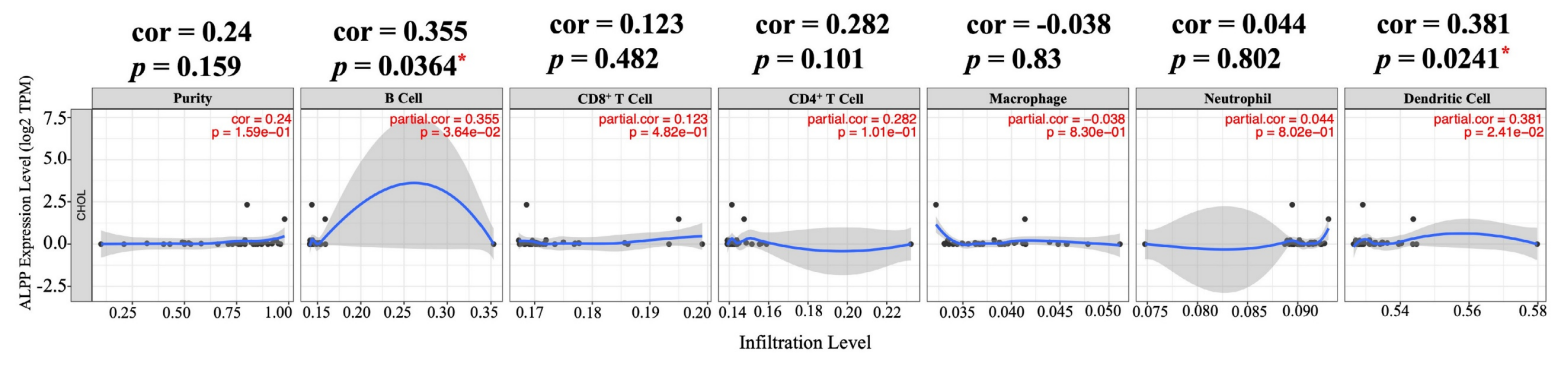
Correlation of *ALPP* expression with immune infiltration level in CCA analyze using TIMER. * p < 0.05.

With the emergence of immune checkpoint inhibitors and immunotherapy, there has been increasing focus on the composition of immune cells within the TME. Understanding the interactions between tumors and immune cells, as well as identifying key immune factors, holds potential for advancing cancer treatments. In this study, we utilized TIMER, a comprehensive web-based tool, to examine the relationship between *ALPP* expression and immune infiltration in CCA 26. We selected *ALPP* expression levels that were positively correlated with tumor purity. Our analysis revealed that *ALPP* expression was significantly positively correlated with the infiltration of B cells (r = 0.355, p = 0.0364) and dendritic cells (DCs) (r = 0.381, p = 0.0241) in CCA (Figure 3). These findings suggest that *ALPP* is associated with immune cell infiltration in CCA.

### 3.3. Construction of co-expression modules of low and high *ALPP* expression in cholangiocarcinoma by WGCNA

**Figure 4:**
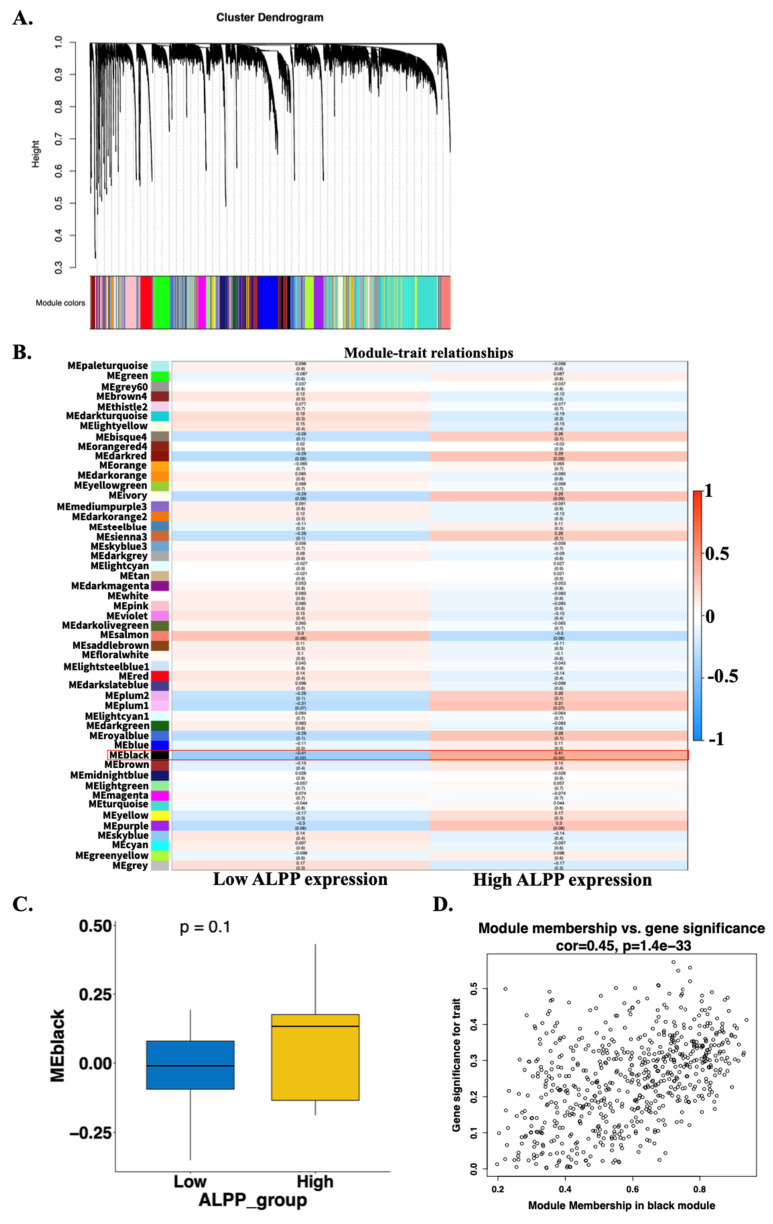
Construction of gene co-expression modules using WGCNA based on TCGA-CHOL RNA-seq data. (A) Gene clustering dendrogram based on topological overlap matrix (TOM) dissimilarity, with module colors assigned using dynamic tree cutting. (B) Heatmap showing the correlation between module eigengenes and clinical traits stratified by low and high *ALPP* expression. (C) Scatter plot illustrating the correlation between gene significance and module membership in the black module.

RNA-seq data were obtained from the TCGA-CHOL dataset available through the UCSC Xena database. The expression values were normalized using a log₂ (RSEM + 1) transformation. Genes with zero variance across all samples were filtered out, resulting in a final dataset containing 19,642 genes. One evident outlier sample (TCGA-W5-AA2H-01), identified through principal component analysis and hierarchical clustering based on gene expression profiles, was excluded from further analysis (Supplementary Figure 1A-D). A weighted gene co-expression network was subsequently constructed using the WGCNA algorithm based on the remaining 35 tumor samples. The Pearson correlation matrix was transformed into an adjacency matrix using a soft-thresholding power of β = 5, which satisfied the scale-free topology criterion (R² = 0.85) (Supplementary Figure 1E-F). A total of 51 co-expression gene modules were identified (Figure 4A). To determine modules most relevant to clinical features, particularly *ALPP* expression, we calculated the correlation coefficients between module eigengenes and traits, along with corresponding p-values. A heatmap was generated to visualize the strength and direction of these correlations. The black module showed the strongest association with *ALPP* expression levels, with significant correlations observed for both low (r = -0.42, p = 0.02) and high (r = 0.42, p = 0.02) expression groups (Figure 4B). Additionally, boxplots were used to compare module eigengene values across *ALPP* expression groups. However, the boxplot comparison for the black module did not reach statistical significance (Figure 4C and Supplementary Figure 2), possibly due to the influence of a small subset of key genes involved in tumor progression. Furthermore, correlation scatter plots were generated to evaluate the relationships among gene significance (GS), module membership (MM), and the *ALPP* phenotype. In the black module, gene significance was significantly correlated with module membership (r = 0.45, p = 1.4e-33) (Figure 4D and Supplementary Figure 3). Based on these findings, the black module was selected for further investigation with *ALPP*-associated biological processes and clinical relevance.

### 3.4. Functional enrichment of the black module reveals potential signaling pathways via GO and KEGG analyses

**Figure 5:**
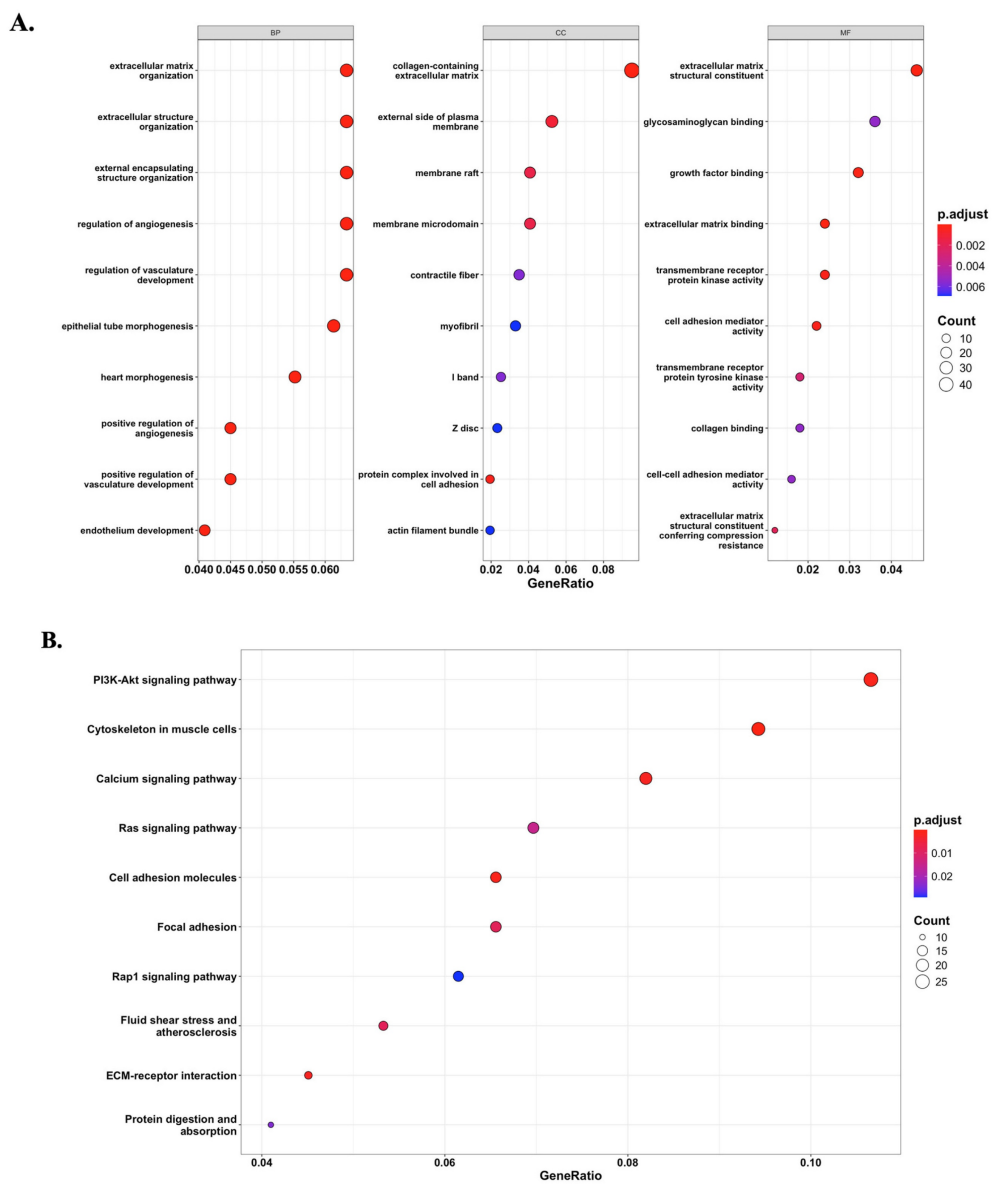
Potential signaling pathways associated with *ALPP* expression phenotype. (A) GO enrichment analysis revealed significantly enriched pathways between *ALPP* high and low expression groups. (B) KEGG analysis identified distinct signaling pathways associated with differential *ALPP* expression.

Based on the module-trait relationship analysis, the black module was found to be significantly associated with *ALPP* expression. To further investigate the biological significance of this module, GO and KEGG enrichment analyses were performed on the 647 genes within the black module. The enrichment results revealed that genes in the black module were predominantly involved in extracellular matrix (ECM)-related pathways (Figure 5A) and the PI3K-Akt signaling pathway (Figure 5B). These findings suggest that *ALPP* may be involved in the regulation of ECM organization and PI3K-Akt signaling, indicating its potential role in modulating TME and cell signaling processes.

### 3.5. *ALPP* methylation in CCA

**Figure 6:**
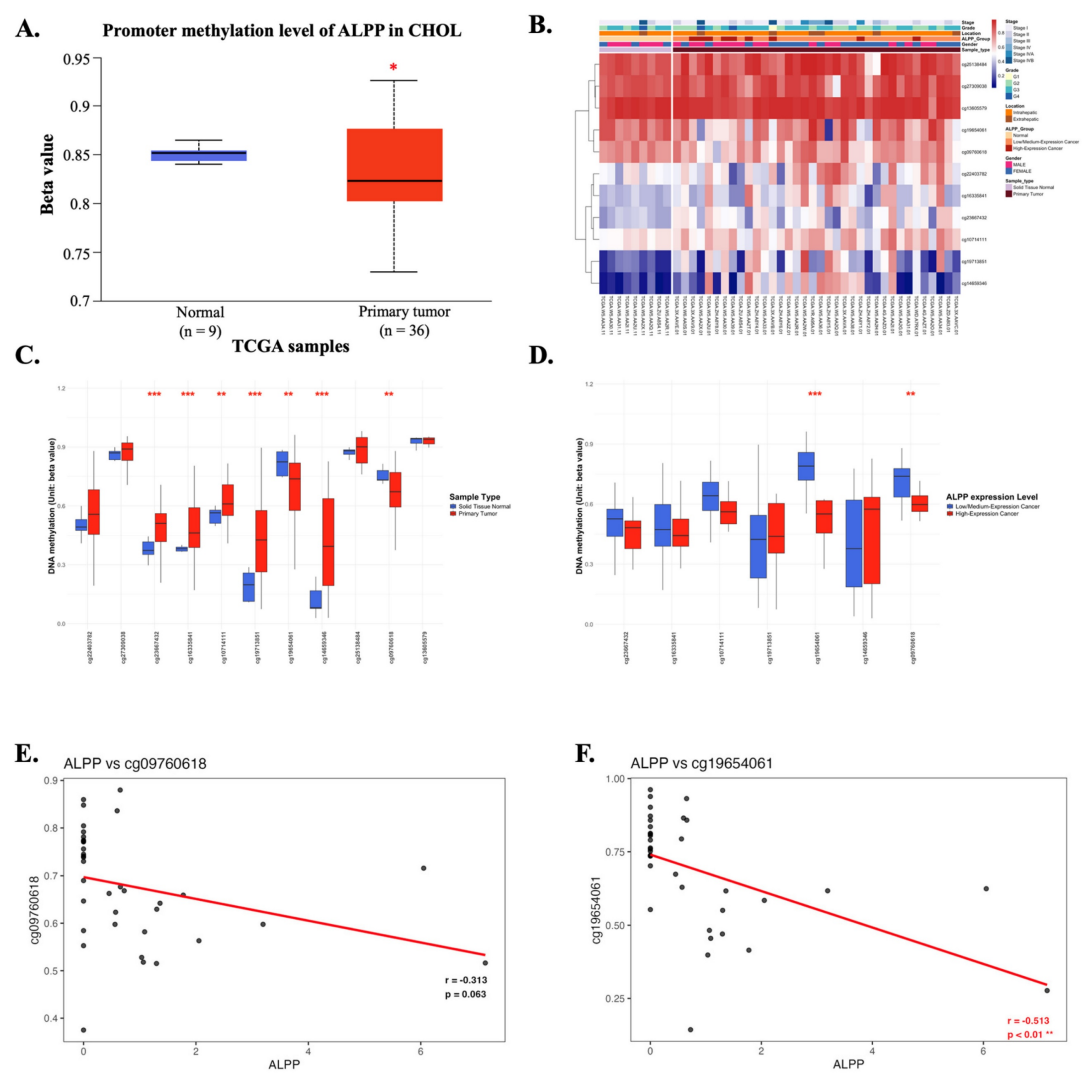
The DNA methylation of *ALPP* in CCA of TCGA (A) Promoter methylation levels of *ALPP* between Normal tissue and CCA. (B) Heatmap of DNA methylation expression level of *ALPP* gene in CCA. (C-D) The beta value of DNA methylation compares Normal tissue with CCA and further compare Low with High *ALPP* level in different single CpG site of the *ALPP* gene in CCA. (E-F) Pearson correlation was used to analyze the association between *ALPP* expression and the methylation levels of the single CpG sites cg09760618 and cg19654061. The threshold of significance was Welch's t-test p value. *<0.05, ** < 0.01, *** <0.001.

DNA methylation is a critical epigenetic modification that plays a significant role in cancer progression 29. Promoter methylation analysis of *ALPP* revealed its potential regulatory role in CCA. In CCA patients, the promoter region of *ALPP* exhibited significantly lower methylation levels compared to normal controls (Figure 6A), suggesting possible epigenetic deregulation. A heatmap analysis was performed to illustrate the clustering patterns of DNA methylation profiles and *ALPP* expression levels in CCA samples (Figure 6B). The methylation status of individual CpG sites was further compared between tumor and normal tissues, as well as in relation to *ALPP* expression (Figure 6C-D). Notably, specific CpG sites, such as cg09760618 and cg19654061, were highlighted in the analysis, with cg19654061 demonstrating a significant negative correlation with *ALPP* expression (Figures 6E-F). These findings suggest that promoter hypomethylation may contribute to the upregulation of *ALPP* in CCA.

### 3.6 *ALPP* is a potential prognostic biomarker in CCA

**Figure 7:**
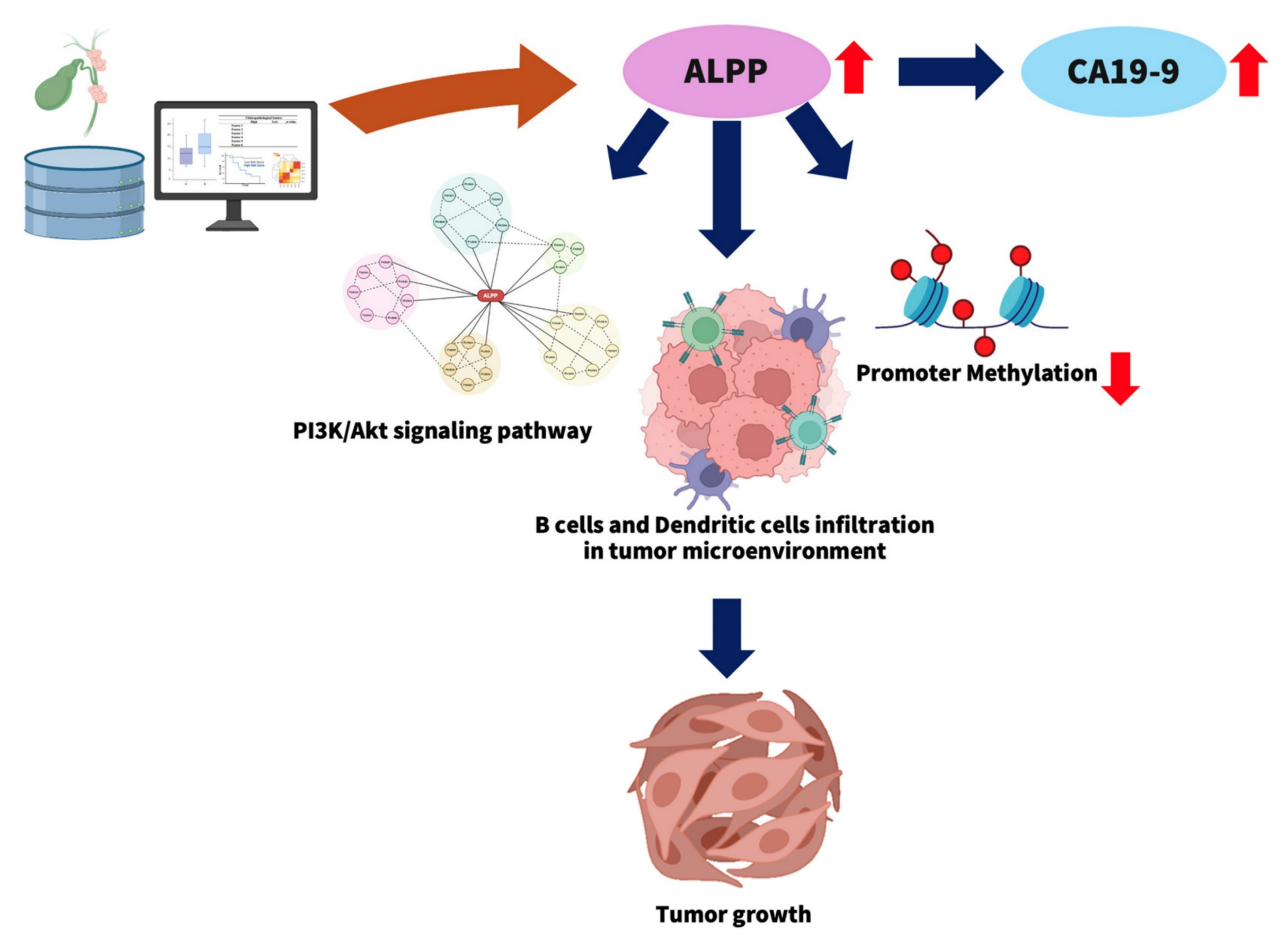
A Database Analysis Model for Investigating *ALPP* Expression in CCA. The figure was created using BioRender (https://app.biorender.com).

This study explores the potential role of elevated *ALPP* expression in the progression of CCA (Figure 7). High *ALPP* expression was found to be associated with increased serum levels of the tumor marker CA19-9, enhanced infiltration of immune cells, including B cells and dendritic cells, and potential activation of the PI3K-Akt signaling pathway. These factors may act synergistically to promote CCA progression and are collectively associated with a poorer prognosis in CCA patients.

## 4. Discussion

ALPP (alkaline phosphatase, placental type) is an isoenzyme of alkaline phosphatase (ALP), first identified by Martin et al. in 1987 and primarily expressed in human placental tissue 30. Accumulating evidence has since demonstrated that ALPP plays a role in modulating both innate and adaptive immune responses, particularly within syncytiotrophoblasts and primordial germ cells, with expression initiating in the early stages of gestation and progressively increasing throughout pregnancy. Structurally, ALPP is a glycosylated, membrane-bound dimeric enzyme anchored to the cell surface via a glycosylphosphatidylinositol (GPI) linkage. Notably, ALPP can also be released from the cell membrane as a secreted protein through the action of specific phospholipases 21. In addition to its physiological roles, ALPP has been identified as a biomarker in various germ cell tumors, including seminomas and dysgerminomas 31, 32. However, the molecular mechanisms underlying involvement of ALPP in tumor progression, as well as its prognostic significance, particularly in CCA, remain largely undefined.

In this study, we first compared the expression profiles of ALPP with other alkaline phosphatase isoenzymes in patients with CCA and found that ALPP may play a central role in CCA progression. Additionally, we observed a positive association between ALPP expression and serum CA19-9 levels, suggesting a potential link between ALPP and established clinical biomarkers. The TME, comprising immune cells, fibroblasts, endothelial cells, extracellular matrix components, blood vessels, and soluble factors, is increasingly recognized as a critical factor in cancer progression and therapeutic response. Emerging evidence highlights the TME as a promising target for the development of effective anticancer therapies 33. Notably, tumors with high ALPP showed reduced cytotoxic T-cell markers and elevated immune checkpoint ligand expression, indicative of immune evasion. This finding aligns with reports that ALPP overexpression can dampen anti-tumor immunity 21, thereby potentially enabling tumor immune escape. In line with this, our TIMER analysis revealed a significant positive correlation between ALPP expression and immune cell infiltration, particularly B cells and dendritic cells. These findings raise the possibility that the prognostic value of ALPP in CCA may be mediated, at least in part, through its interaction with the immune microenvironment. Interestingly, although tumor-infiltrating B lymphocytes are often linked to better outcomes in cholangiocarcinoma and related biliary cancers 34, the co-occurrence of high ALPP expression with increased B cell/DC infiltration in our study suggests a complex immune context. One hypothesis is that ALPP may drive an immunosuppressive microenvironment despite the presence of immune cells. ALPP's enzymatic activity could modulate extracellular adenosine or phosphate metabolites to dampen immune activation 21. Additionally, ALPP-overexpressing tumors might recruit immunoregulatory subsets of immune cells. For example, placental ALP has immunomodulatory properties that could promote tolerogenic dendritic cells. An increase in plasmacytoid dendritic cells, which are associated with poorer survival in solid tumors 35, in ALPP-high CCA tumors could impair effective anti-tumor immunity. Likewise, B cells in this context might be skewed toward regulatory phenotypes. These possibilities suggest that ALPP contributes to immune evasion by qualitatively altering the tumor-infiltrating immune cells. Our findings suggest that ALPP-high cholangiocarcinoma tumors could be fostering an immunosuppressive microenvironment. This aligns with reports that most intrahepatic cholangiocarcinomas are 'cold' tumors with scant T-cell infiltration due to immune evasion strategies 36. Prior studies have shown that elevated ALPP can impair macrophage phagocytic activity and T-cell mediated responses 21, supporting the notion that ALPP helps tumors escape immune surveillance. Therefore, ALPP might indirectly promote tumor progression by attenuating anti-tumor immunity. In light of this, ALPP is not only a biomarker but also a potential therapeutic target; indeed, placental alkaline phosphatase is being explored as a target for immunotherapy in other cancers 22. Targeting ALPP could hypothetically reduce immune evasion and render the tumor more susceptible to immunotherapy. However, whether the impact of ALPP on patient outcomes is directly linked to B cell and dendritic cell activity warrants further mechanistic investigation.

To further elucidate the functional role of ALPP in CCA, we performed a WGCNA to identify gene modules associated with low and high ALPP expression. This approach enabled the identification of a module of interest, which was subsequently subjected to GO and KEGG enrichment analyses. The results revealed significant enrichment in extracellular matrix-related pathways and activation of the PI3K-Akt signaling pathway. The PI3K-Akt pathway is a well-conserved signaling cascade in eukaryotic cells, known to promote cell survival, proliferation, and cell cycle progression. Meanwhile, extracellular matrix components are recognized as key mediators of tumor cell migration and metastasis 37, 38. Notably, previous studies have implicated alkaline phosphatase isoenzymes in modulating the PI3K-Akt signaling pathway 39, 40. While our data demonstrate a strong association between ALPP overexpression and elevated PI3K-Akt pathway activity, direct causation has not been definitively proven in this study. Our results are consistent with the known importance of PI3K-Akt signaling in cholangiocarcinoma progression 41; thus, it is plausible that ALPP contributes to tumor growth through this pathway. One hypothesis is that ALPP's enzymatic activity might lead to microenvironmental changes (e.g., in phosphate-related metabolites or inflammatory mediators) that secondarily activate PI3K/Akt signaling. However, we acknowledge this linkage as a potential mechanism rather than a confirmed fact. Further functional experiments (such as ALPP knockdown or overexpression followed by phospho-Akt monitoring) would be required to establish a direct causal relationship. We have revised the text to describe ALPP's effect on immune evasion and PI3K/Akt activation as an association supported by our data, in line with mechanistic insights from the literature, without implying unwarranted causality. Taken together, these findings suggest that ALPP may contribute to CCA progression by regulating extracellular matrix remodeling and activation of PI3K-Akt signaling, thus offering new insights into its functional role in tumor biology.

DNA methylation plays a critical role in tumorigenesis and cancer progression and is particularly relevant in CCA 42. Given the marked genetic heterogeneity of CCA, this malignancy poses unique therapeutic challenges, thereby driving interest in the clinical utility of DNA methylation as a tool for prognostic biomarker discovery and therapeutic stratification 43. In the present study, we further explored the methylation status of ALPP in CCA and its association with patient prognosis. Our findings suggest that CCA may exploit distinct methylation sites within the ALPP gene to influence tumor progression, highlighting the potential of ALPP methylation as a novel epigenetic marker in CCA.

This study has several limitations. First, our analyses are based on 45 TCGA‑CHOL tumours, a number comparable to prior TCGA‑CHOL studies yet inherently under‑powered for some statistical tests (e.g., subgroup analyses). Because the dataset represents a single retrospective cohort, cohort‑specific biases cannot be excluded. Consequently, our results should be viewed as hypothesis‑generating until they are verified in larger, prospectively collected, multi‑centre series. Second, the results, derived from retrospective public data, are correlative and do not establish causation. Additionally, this study lacked experimental validation of ALPP's functional role. Therefore, caution is warranted in interpreting ALPP as a prognostic biomarker until further evidence is obtained. Future studies should validate the prognostic significance of ALPP in larger, independent CCA cohorts and investigate the mechanistic underpinnings of ALPP in cholangiocarcinoma. For instance, prospective tissue microarray analyses or multi-center cohort studies could confirm the association between ALPP expression and patient outcomes. Likewise, in vitro and in vivo functional assays (e.g., ALPP knockdown in CCA cell lines or xenograft models) will be important to determine whether ALPP actively drives tumor progression or immune modulation. Such follow-up studies, combined with external dataset validations 44, will solidify the clinical relevance of ALPP and explore its potential as a therapeutic target in CCA.

## 5. Conclusions

In summary, our study demonstrates that ALPP expression is significantly associated with immune cell infiltration, particularly B cells and dendritic cells. Furthermore, ALPP may regulate critical signaling pathways in CCA, including the activation of the PI3K-Akt signaling pathway, thereby potentially contributing to tumor progression. To the best of our knowledge, this is the first study to uncover a strong association between ALPP expression and CCA. Collectively, these findings suggest that ALPP may function as an independent prognostic biomarker and is closely linked to the TME in CCA.

## Supplementary Material

Supplementary figures.

## Figures and Tables

**Table 1:**
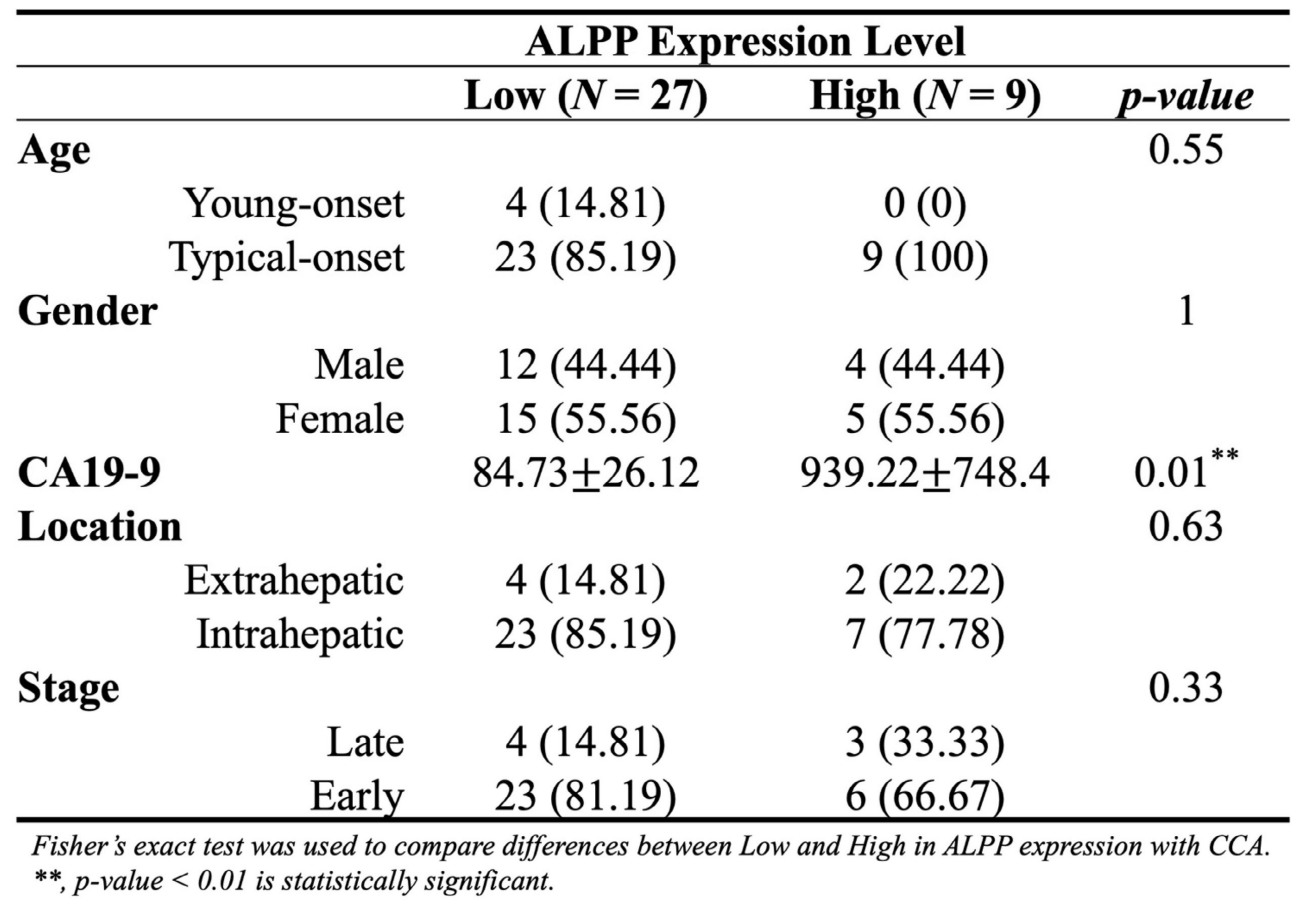
Distribution of demographic characteristics in CCA patients with low (n = 27) and high (n = 9) ALPP expression

**Table 2:**
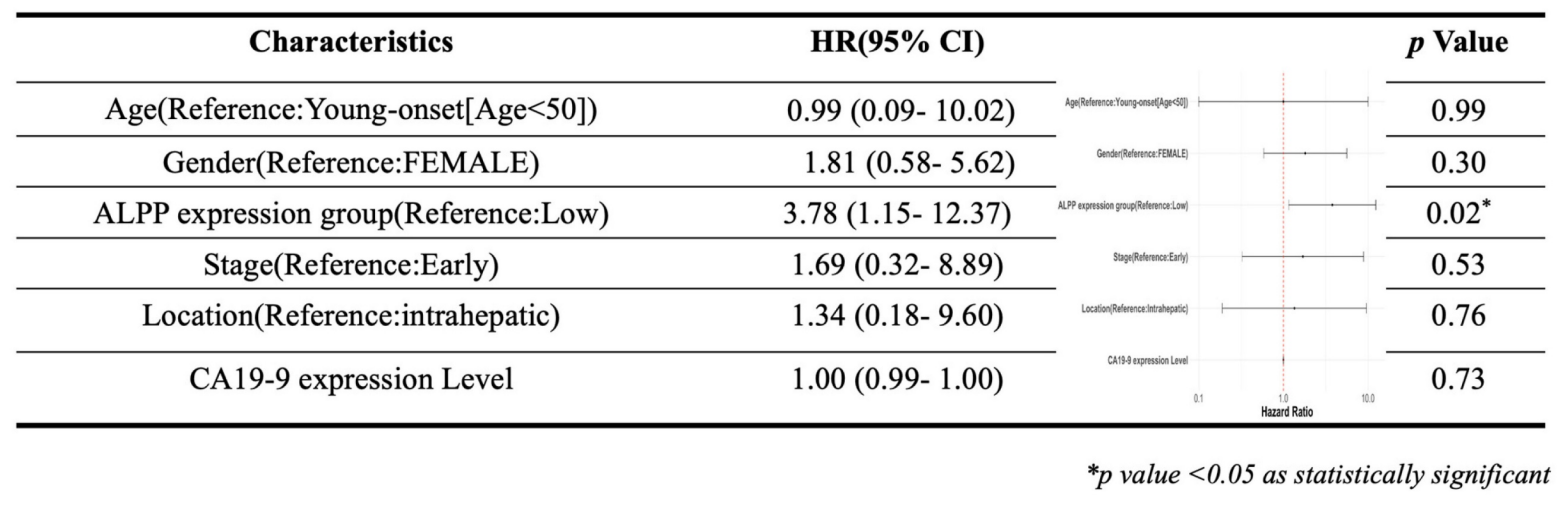
Multivariate Cox regression analysis of clinicopathological features (including ALPP expression) with OS in the TCGA datasets
