# Supplementary material for: Aberrant ALPP Expression Serves as a Prognostic Biomarker and Facilitates Cholangiocarcinoma Progression through Immune Evasion and PI3K-Akt Signaling Activation
Source: Int J Med Sci. 2025 Oct 1;22(16):4214–26. doi: 10.7150/ijms.116260 (PMC12595322; doi:10.7150/ijms.116260)
Supplement: Supplementary file 1 — Supplementary figures. [file ijmsv22p4214s1.pdf]

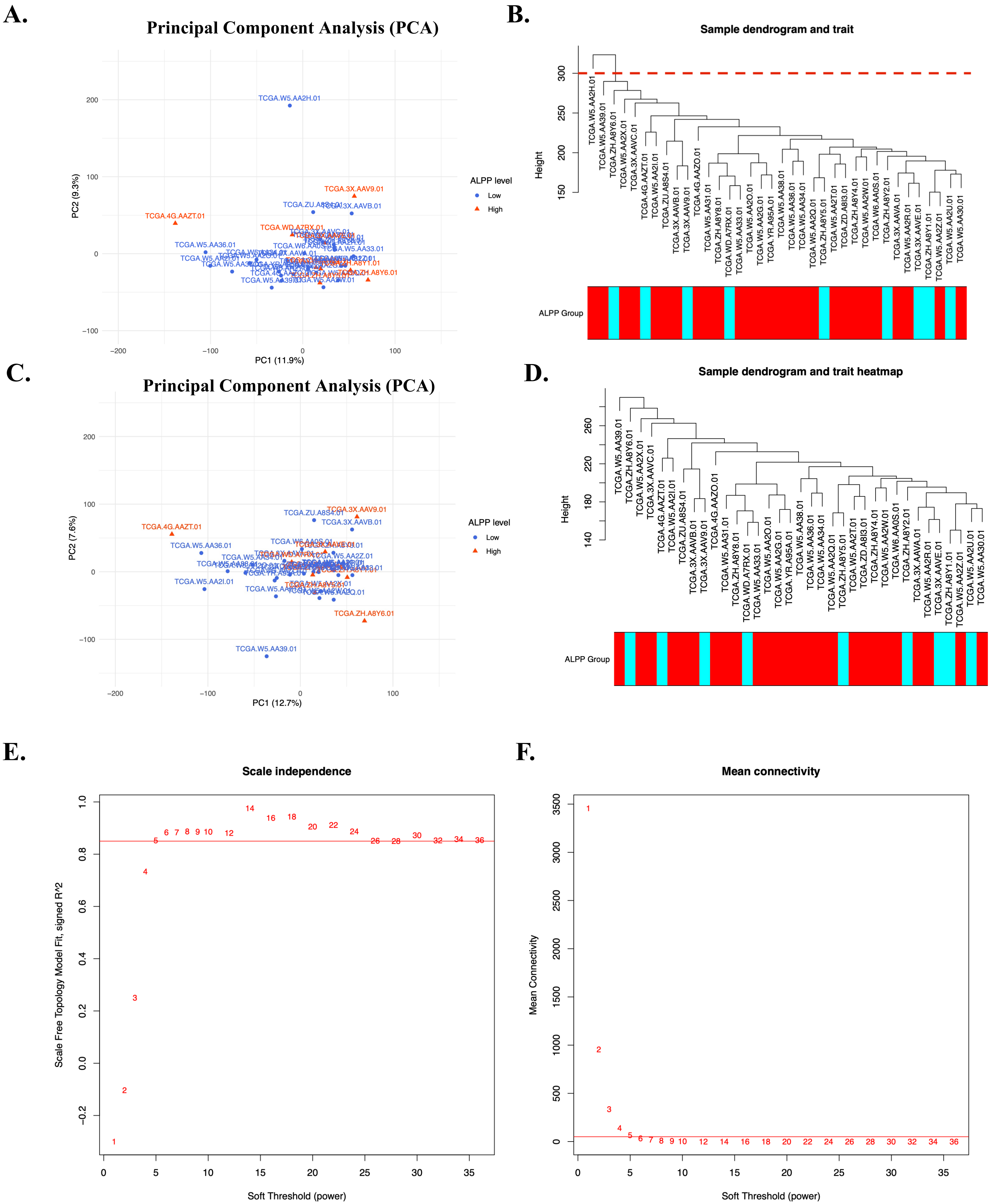
A.
Principal Component Analysis (PCA)
PC1 (11.9%)
PC2 (9.3%)
ALPP level
Low
High
B.
Sample dendrogram and trait
Height
ALPP Group
C.
Principal Component Analysis (PCA)
PC1 (12.7%)
PC2 (7.6%)
ALPP level
Low
High
D.
Sample dendrogram and trait heatmap
Height
ALPP Group
E.
Scale independence
Scale Free Topology Model Fit, signed R^2
Soft Threshold (power)
F.
Mean connectivity
Mean Connectivity
Soft Threshold (power)

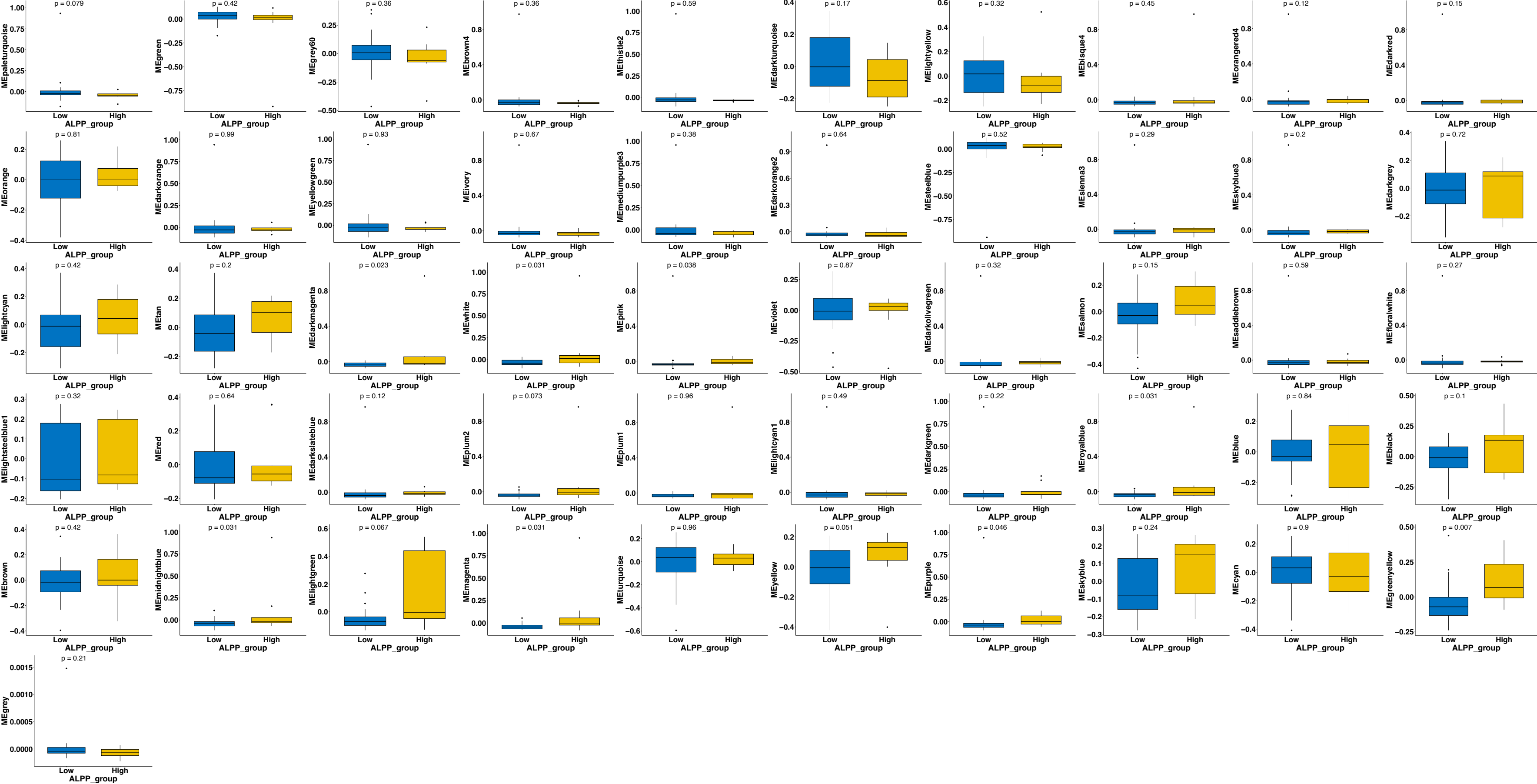

p = 0.079
MEpaleturquoise
p = 0.42
MEgreen
p = 0.36
MEgrey60
p = 0.36
MEbrown4
p = 0.59
MEthistle2
p = 0.17
MEdarkturquoise
p = 0.32
MElightyellow
p = 0.45
MEbisque4
p = 0.12
MEorangered4
p = 0.15
MEdarkred
p = 0.81
MEorange
p = 0.99
MEdarkorange
p = 0.93
MEyellowgreen
p = 0.67
MEivory
p = 0.38
MEmediumpurple3
p = 0.64
MEdarkorange2
p = 0.52
MEsteelblue
p = 0.29
MEsienna3
p = 0.2
MEskyblue3
p = 0.72
MEdarkgrey
p = 0.42
MElightcyan
p = 0.2
MEtan
p = 0.023
MEdarkmagenta
p = 0.031
MEwhite
p = 0.038
MEpink
p = 0.87
MEviolet
p = 0.32
MEdarkolivegreen
p = 0.15
MEsalmon
p = 0.59
MEsaddlebrown
p = 0.27
MEfloralwhite
p = 0.32
MElightsteelblue1
p = 0.64
MEred
p = 0.12
MEdarkslateblue
p = 0.073
MEplum2
p = 0.96
MEplum1
p = 0.49
MElightcyan1
p = 0.22
MEdarkgreen
p = 0.031
MEroyalblue
p = 0.84
MEblue
p = 0.1
MEblack
p = 0.42
MEbrown
p = 0.031
MEmidnightblue
p = 0.067
MElightgreen
p = 0.031
MEmagenta
p = 0.96
MEturquoise
p = 0.051
MEyellow
p = 0.046
MEpurple
p = 0.24
MEskyblue
p = 0.9
MEcyan
p = 0.007
MEgreenyellow
p = 0.21
MEgrey
Low
High
ALPP_group

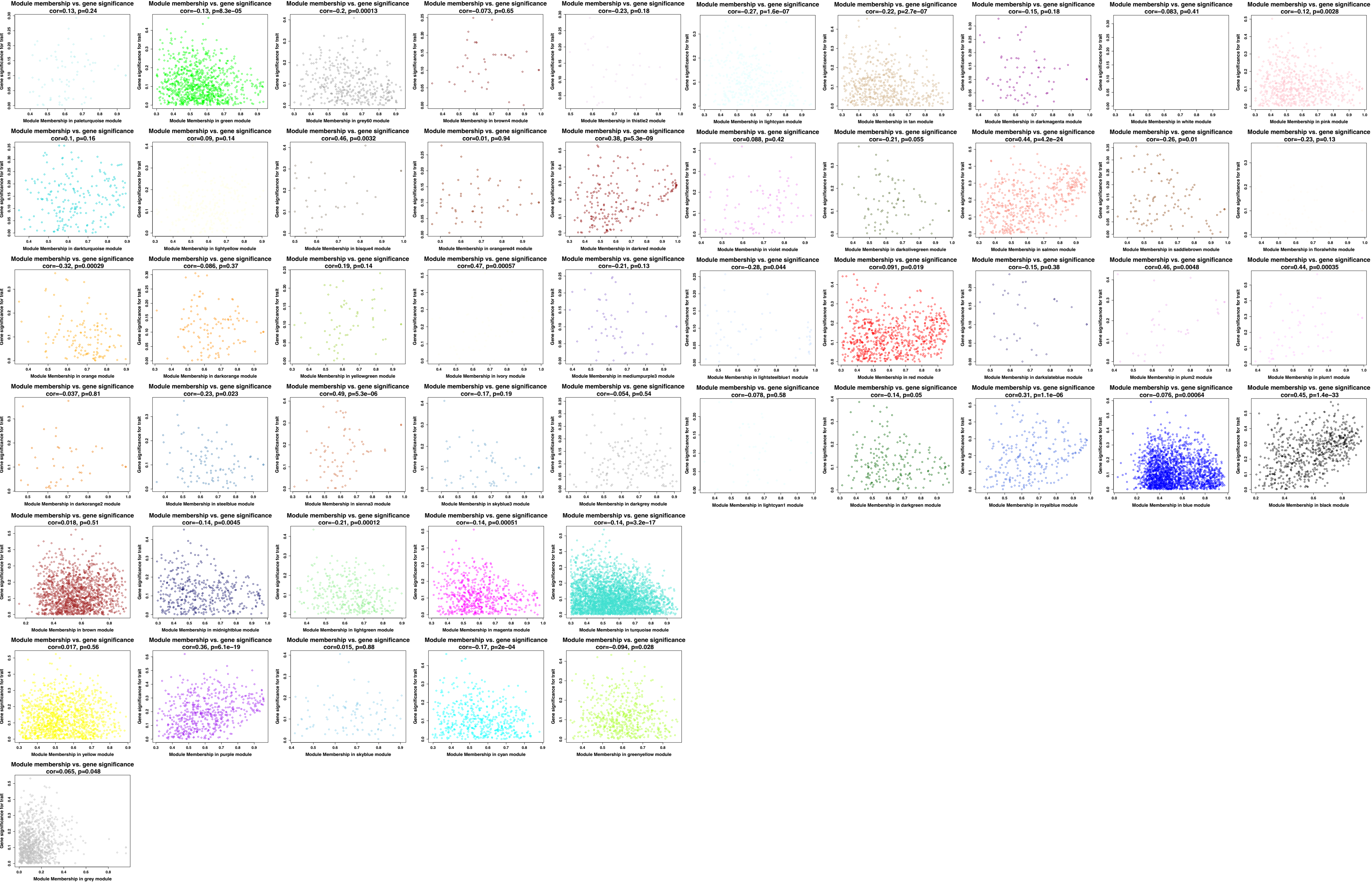
Module membership vs. gene significance
cor=0.13, p=0.24
Gene significance for trait
Module Membership in paleturquoise module
Module membership vs. gene significance
cor=−0.13, p=8.3e−05
Gene significance for trait
Module Membership in green module
Module membership vs. gene significance
cor=−0.2, p=0.00013
Gene significance for trait
Module Membership in grey60 module
Module membership vs. gene significance
cor=−0.073, p=0.65
Gene significance for trait
Module Membership in brown4 module
Module membership vs. gene significance
cor=−0.23, p=0.18
Gene significance for trait
Module Membership in thistle2 module
Module membership vs. gene significance
cor=−0.27, p=1.6e−07
Gene significance for trait
Module Membership in lightcyan module
Module membership vs. gene significance
cor=−0.22, p=2.7e−07
Gene significance for trait
Module Membership in tan module
Module membership vs. gene significance
cor=−0.15, p=0.18
Gene significance for trait
Module Membership in darkmagenta module
Module membership vs. gene significance
cor=−0.083, p=0.41
Gene significance for trait
Module Membership in white module
Module membership vs. gene significance
cor=−0.12, p=0.0028
Gene significance for trait
Module Membership in pink module
Module membership vs. gene significance
cor=0.1, p=0.16
Gene significance for trait
Module Membership in darkturquoise module
Module membership vs. gene significance
cor=0.09, p=0.14
Gene significance for trait
Module Membership in lightyellow module
Module membership vs. gene significance
cor=0.46, p=0.0032
Gene significance for trait
Module Membership in bisque4 module
Module membership vs. gene significance
cor=0.01, p=0.94
Gene significance for trait
Module Membership in orangered4 module
Module membership vs. gene significance
cor=0.38, p=5.3e−09
Gene significance for trait
Module Membership in darkred module
Module membership vs. gene significance
cor=0.088, p=0.42
Gene significance for trait
Module Membership in violet module
Module membership vs. gene significance
cor=−0.21, p=0.055
Gene significance for trait
Module Membership in darkolivegreen module
Module membership vs. gene significance
cor=0.44, p=4.2e−24
Gene significance for trait
Module Membership in salmon module
Module membership vs. gene significance
cor=−0.26, p=0.01
Gene significance for trait
Module Membership in saddlebrown module
Module membership vs. gene significance
cor=−0.23, p=0.13
Gene significance for trait
Module Membership in floralwhite module
Module membership vs. gene significance
cor=−0.32, p=0.00029
Gene significance for trait
Module Membership in orange module
Module membership vs. gene significance
cor=−0.086, p=0.37
Gene significance for trait
Module Membership in darkorange module
Module membership vs. gene significance
cor=0.19, p=0.14
Gene significance for trait
Module Membership in yellowgreen module
Module membership vs. gene significance
cor=0.47, p=0.00057
Gene significance for trait
Module Membership in ivory module
Module membership vs. gene significance
cor=−0.21, p=0.13
Gene significance for trait
Module Membership in mediumpurple3 module
Module membership vs. gene significance
cor=−0.28, p=0.044
Gene significance for trait
Module Membership in lightsteelblue1 module
Module membership vs. gene significance
cor=0.091, p=0.019
Gene significance for trait
Module Membership in red module
Module membership vs. gene significance
cor=−0.15, p=0.38
Gene significance for trait
Module Membership in darkslateblue module
Module membership vs. gene significance
cor=0.46, p=0.0048
Gene significance for trait
Module Membership in plum2 module
Module membership vs. gene significance
cor=0.44, p=0.00035
Gene significance for trait
Module Membership in plum1 module
Module membership vs. gene significance
cor=−0.037, p=0.81
Gene significance for trait
Module Membership in darkorange2 module
Module membership vs. gene significance
cor=−0.23, p=0.023
Gene significance for trait
Module Membership in steelblue module
Module membership vs. gene significance
cor=0.49, p=5.3e−06
Gene significance for trait
Module Membership in sienna3 module
Module membership vs. gene significance
cor=−0.17, p=0.19
Gene significance for trait
Module Membership in skyblue3 module
Module membership vs. gene significance
cor=−0.054, p=0.54
Gene significance for trait
Module Membership in darkgrey module
Module membership vs. gene significance
cor=−0.078, p=0.58
Gene significance for trait
Module Membership in lightcyan1 module
Module membership vs. gene significance
cor=−0.14, p=0.05
Gene significance for trait
Module Membership in darkgreen module
Module membership vs. gene significance
cor=0.31, p=1.1e−06
Gene significance for trait
Module Membership in royalblue module
Module membership vs. gene significance
cor=−0.076, p=0.00064
Gene significance for trait
Module Membership in blue module
Module membership vs. gene significance
cor=0.45, p=1.4e−33
Gene significance for trait
Module Membership in black module
Module membership vs. gene significance
cor=0.018, p=0.51
Gene significance for trait
Module Membership in brown module
Module membership vs. gene significance
cor=−0.14, p=0.0045
Gene significance for trait
Module Membership in midnightblue module
Module membership vs. gene significance
cor=−0.21, p=0.00012
Gene significance for trait
Module Membership in lightgreen module
Module membership vs. gene significance
cor=−0.14, p=0.00051
Gene significance for trait
Module Membership in magenta module
Module membership vs. gene significance
cor=−0.14, p=3.2e−17
Gene significance for trait
Module Membership in turquoise module
Module membership vs. gene significance
cor=0.017, p=0.56
Gene significance for trait
Module Membership in yellow module
Module membership vs. gene significance
cor=0.36, p=6.1e−19
Gene significance for trait
Module Membership in purple module
Module membership vs. gene significance
cor=0.015, p=0.88
Gene significance for trait
Module Membership in skyblue module
Module membership vs. gene significance
cor=−0.17, p=2e−04
Gene significance for trait
Module Membership in cyan module
Module membership vs. gene significance
cor=−0.094, p=0.028
Gene significance for trait
Module Membership in greenyellow module
Module membership vs. gene significance
cor=0.065, p=0.048
Gene significance for trait
Module Membership in grey module
